# Exploring the causal associations of gout and serum uric acid levels on erectile dysfunction: A Mendelian randomization study

**DOI:** 10.1097/MD.0000000000041679

**Published:** 2025-02-21

**Authors:** Jianhua Zhang, Lei Ji

**Affiliations:** a Department of Orthopaedics, Nantong Second People’s Hospital, Nantong City, China.

**Keywords:** causal association, erectile dysfunction, gout, Mendelian randomization, serum uric acid

## Abstract

Several observational studies have suggested a possible link between gout, serum uric acid (UA) levels, and erectile dysfunction (ED). Nonetheless, the current body of evidence does not allow for a conclusive determination regarding the influence of gout and serum UA on the likelihood of developing ED. The primary aim of this research was to explore the potential causal relationship between gout and serum UA levels in relation to ED utilizing Mendelian randomization (MR) analysis. The principal analytical method employed was inverse variance weighting (IVW). Following this, a sensitivity analysis was performed using Cochran *Q*-test, funnel plots, MR-Egger regression, and the leave-one-out method. The findings from the IVW analysis revealed no significant association between gout and ED (odds ratio [OR] = 1.004, 95% confidence interval [CI]: 0.948–1.063, *P* = .888), nor between serum UA levels and ED (OR = 1.013, 95% CI: 0.775–2.126, *P* = .333). The results from the supplementary methods corroborated those obtained from the IVW approach. This study confirmed the absence of heterogeneity and horizontal pleiotropy, with consistent results across all sensitivity analyses. The MR analysis did not yield genetic-level evidence to substantiate a direct causal relationship between gout, serum UA, and ED.

## 1. Introduction

Erectile dysfunction (ED) is a prevalent condition among men, defined by the persistent or recurrent inability to achieve and sustain an erection adequate for satisfactory sexual intercourse. This condition can significantly impact the quality of life of affected individuals and their familial relationships.^[1]^ Empirical evidence suggests that the prevalence of ED escalates with advancing age, beginning at approximately 10% in men under 50 years, increasing to 20% to 40% in men aged 60 to 69,^[2]^ around 50% in men over 70,^[3]^ and reaching approximately 86% in those over 80.^[4]^ Projections indicate that by 2025, the global prevalence of ED may affect up to 322 million individuals.^[5]^ Consequently, the provision of early treatment and intervention for this condition is of paramount importance. Various factors have been identified as potential contributors to the onset of ED, including age, obesity, smoking, depression, and hypertension. A cross-sectional study has demonstrated that ED is particularly prevalent among men with gout, with a significantly higher proportion of gout patients (63.76%) experiencing ED compared to their counterparts without gout (60.51%). Furthermore, a notably greater percentage of patients with gout (22.26%) exhibit severe ED in contrast to those without the condition (17.15%).^[6]^

Gout represents the most common form of inflammatory arthritis among men, characterized by the accumulation of urate in the joints and adjacent tissues, alongside chronic hyperuricemia.^[7]^ A systematic review conducted by Du et al revealed that individuals diagnosed with gout exhibited a 1.44-fold increased likelihood of developing ED compared to control subjects. Even after controlling for age and comorbid conditions, gout was consistently identified as a significant risk factor for ED.^[8]^ In a similar vein, another systematic review encompassing a larger number of studies indicated that the risk of ED among patients with gout was 1.2 times greater than that of individuals without the condition.^[9]^ Elevated serum uric acid (UA) levels are recognized as a primary risk factor for gout attacks and are closely linked to endothelial dysfunction, oxidative stress, and inflammation, all of which are potential contributors to ED.^[10]^ An experimental model in hyperuricemia rats showed that reduced protein expression of eNOS, p-eNOS, and nNOS and increased ROS in spongy tissues may be 1 of the key mechanisms of ED induced by hyperuricemia.^[11]^ Nonetheless, the degree of association between hyperuricemia and ED remains a contentious issue within clinical research. Tuokko et al presented a contrasting viewpoint, contending that elevated serum UA levels do not constitute an independent risk factor for ED and that predictions regarding ED cannot be made solely based on serum UA levels.^[12]^ Additionally, a cross-sectional study conducted among Chinese men found that elevated serum UA levels acted as an independent protective factor against ED.^[13]^ Consequently, the relationship between gout, UA levels, and the risk of ED continues to be a subject of active scholarly debate.

Erectile dysfunction constitutes a significant public health issue on a global scale. Consequently, it is essential to ascertain whether gout and serum UA levels serve as risk factors for ED, thereby informing strategies for disease prevention. The majority of current research employs observational epidemiological methodologies, which are prone to issues such as reverse causality and confounding variables. To mitigate these concerns, we utilized Mendelian randomization (MR) to elucidate the causal relationship between these 2 variables. MR functions similarly to a randomized controlled trial by employing genetic information as an instrumental variable for exposure, thereby reducing susceptibility to confounding factors and enabling causal inferences while adhering to statistical assumptions.^[14]^ To our knowledge, this study represents the first investigation into the causal relationship between gout and serum UA levels in relation to the risk of ED through MR analysis.

## 2. Methods

### 2.1. GWAS data sources

The data employed in this research were sourced from the IEU GWAS database at the University of Bristol (https://gwas.mrcieu.ac.uk). The dataset pertaining to gout exposure can be retrieved using GWAS ID: finn-b-M13_GOUT, which encompasses 150,797 samples and 16,380,152 single-nucleotide polymorphisms (SNPs). Summary statistics for serum UA levels are available through GWAS ID: ebi-a-GCST90018977,^[15]^ which includes 343,836 samples and 19,041,286 SNPs. Furthermore, summary data concerning ED as an outcome variable can be accessed via GWAS ID: ebi-a-GCST006956,^[16]^ comprising 223,805 samples and 9310,195 SNPs. All participants in these datasets were drawn from European populations. The aforementioned datasets are accessible at https://gwas.mrcieu.ac.uk and were utilized for the MR analysis. A detailed description of the GWAS data corresponding to each phenotype is presented in Table 1. All the data for this study were publicly available summary statistics. Therefore, ethical approval and consent to participate were not required.

**Table 1:** Description of GWAS data used for each phenotype.

Phenotype	GWAS ID	Yr	Sample size	SNPs	Ancestry
Gout	finn-b-M13_GOUT	2021	150,797	16,380,152	European
Serum UA levels	ebi-a-GCST90018977	2021	343,836	19,041,286	European
Erectile dysfunction	ebi-a-GCST006956	2021	223,805	9,310,196	European

GWAS = genome-wide association study, SNP = single-nucleotide polymorphism, UA = uric acid.

### 2.2. Instrumental variables selection process

**Figure 1.**
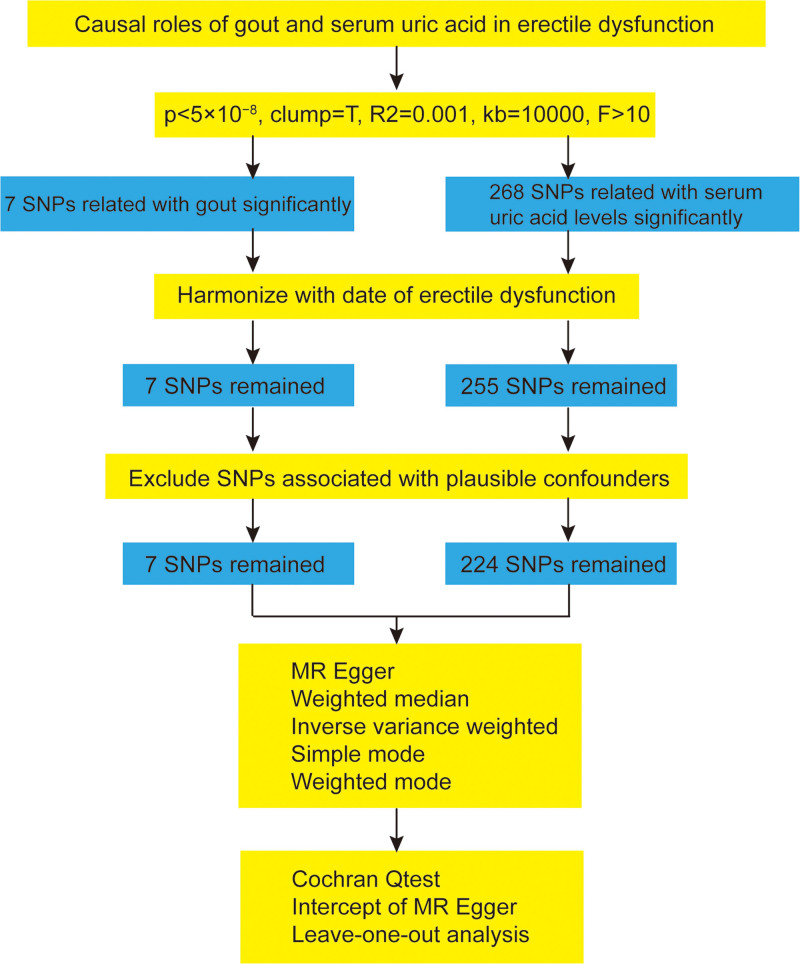
Flowchart for the MR analysis. MR = Mendelian randomization, SNP = single-nucleotide polymorphism.

MR analysis employs SNPs as instrumental variables (IVs) to investigate the causal relationship between exposure and outcome. The selection of IVs in this study adheres to 3 fundamental assumptions:^[14]^ a robust correlation exists between the IVs and the exposure, there is no direct association between the IVs and the outcome, and the IVs are not correlated with confounding variables. The genome-wide significance threshold for serum UA levels, gout, and ED was established at *P* < 5 × 10^−8^. The linkage disequilibrium (*R*^2^) threshold was set at 0.001, with a genetic distance of 10 MB. IVs devoid of linkage effects were selected from the dataset. We consulted the NHGRI-EBI Catalog (https://www.ebi.ac.uk/gwas/) to identify potential confounders, including body mass index, smoking status, blood pressure, and diabetes. SNPs associated with these confounding factors were excluded prior to conducting the MR analysis to mitigate their potential impact on the results. Furthermore, to prevent bias arising from weak IVs, the *F*-statistics of the SNPs were computed using the formula: *F* = [(n − *k* − 1)/*k*]/[*R*^2^/(1 − *R*^2^)].^[17]^ The *F*-statistic values indicate the strength of the IVs, with values <10 typically considered indicative of weak IVs and thus excluded. Ultimately, the SNPs related to exposure and outcome were combined, ensuring the removal of palindromic sequences and incompatible SNPs. A detailed flow chart illustrating this process is presented in Figure 1.

### 2.3. Mendelian randomization

In this research, we utilized the inverse variance weighting (IVW) method as the principal analytical technique to assess the causal relationship between genetically predicted gout and serum UA levels in relation to the risk of ED. In the absence of heterogeneity and pleiotropy, the findings derived from the IVW method are regarded as the most robust.^[18]^ Additional methodologies employed in this analysis include MR-Egger, weighted median, weighted pattern, and simple pattern approaches.

Mendelian randomization-Egger (MR-Egger) is an analysis method for MR using summarized genetic data. The MR-Egger method enables us to assess whether genetic variants have pleiotropic effects on the outcome that differ on average from zero. We utilized the MR-Egger regression to evaluate the pleiotropy of IVs, and *P* < .05 represents having pleiotropy. The heterogeneity between the genetic instruments was evaluated by applying Cochran *Q*-test. Heterogeneity was considered nonexistent when the *P*-value of Cochran *Q* was > .05. we used the Leave-one-out method to verify the robustness of the findings. All the statistical analyses were performed using R software (version 4.4.1, TwoSampleMR package 0.6.6). A significance level of *P* < .05 was considered indicative of a statistically significant result.

## 3. Results

### 3.1. Selection of instrumental variables

Following the selection of SNPs using a *P*-value threshold of *P* < 5 × 10^−8^, we performed a linkage disequilibrium analysis, aligned the coding alleles between the summary statistics of the exposure and outcome, and eliminated SNPs that were associated with potential confounding variables. This methodology enabled us to identify valid IVs that conformed to the 3 essential assumptions of MR. Specifically, we identified 7 SNPs associated with gout and 224 SNPs linked to serum UA levels. In our analysis, the *F*-statistic for each SNP exceeded 10, suggesting a diminished risk of bias attributable to weak instruments. Comprehensive details regarding the SNPs can be found in Table S1, Supplemental Digital Content, http://links.lww.com/MD/O439.

### 3.2. Causal effects of gout on ED

**Figure 2.**
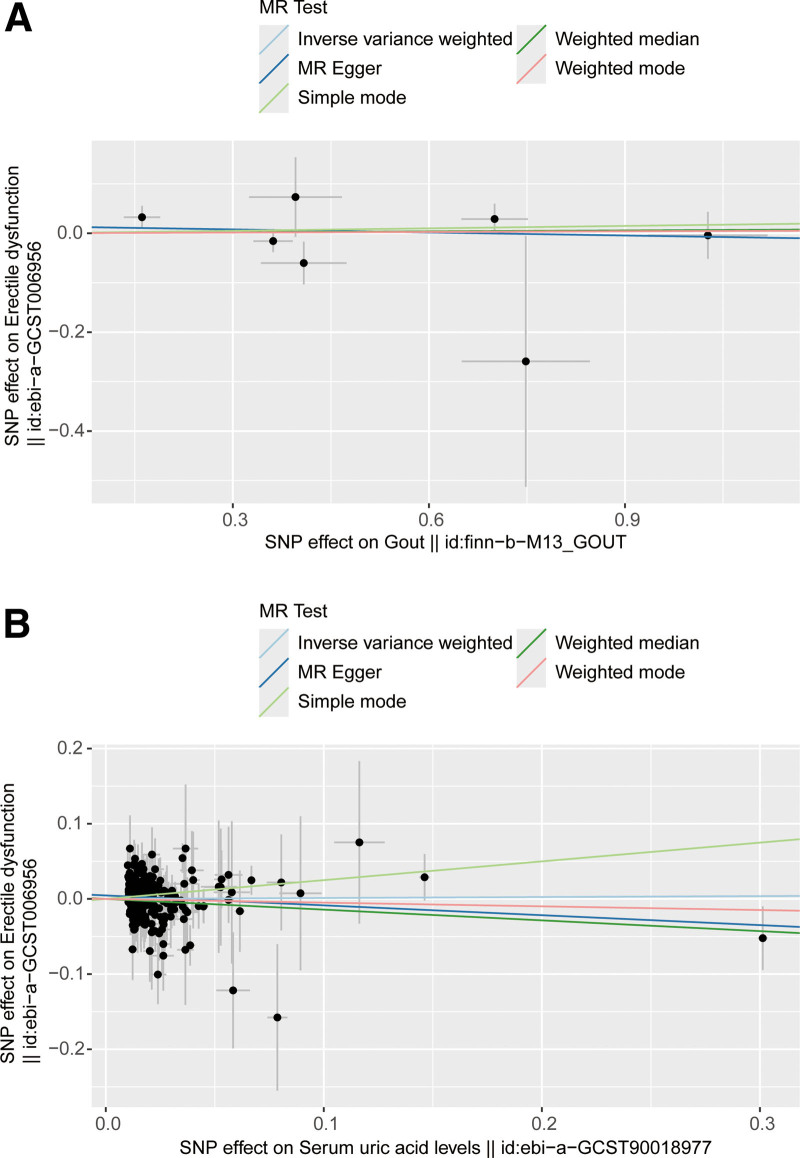
The causality of gout (A) and serum UA levels (B) on ED risk. The slope represents the magnitude of the causal effect. ED = erectile dysfunction, MR = Mendelian randomization, UA = uric acid.

**Figure 3.**
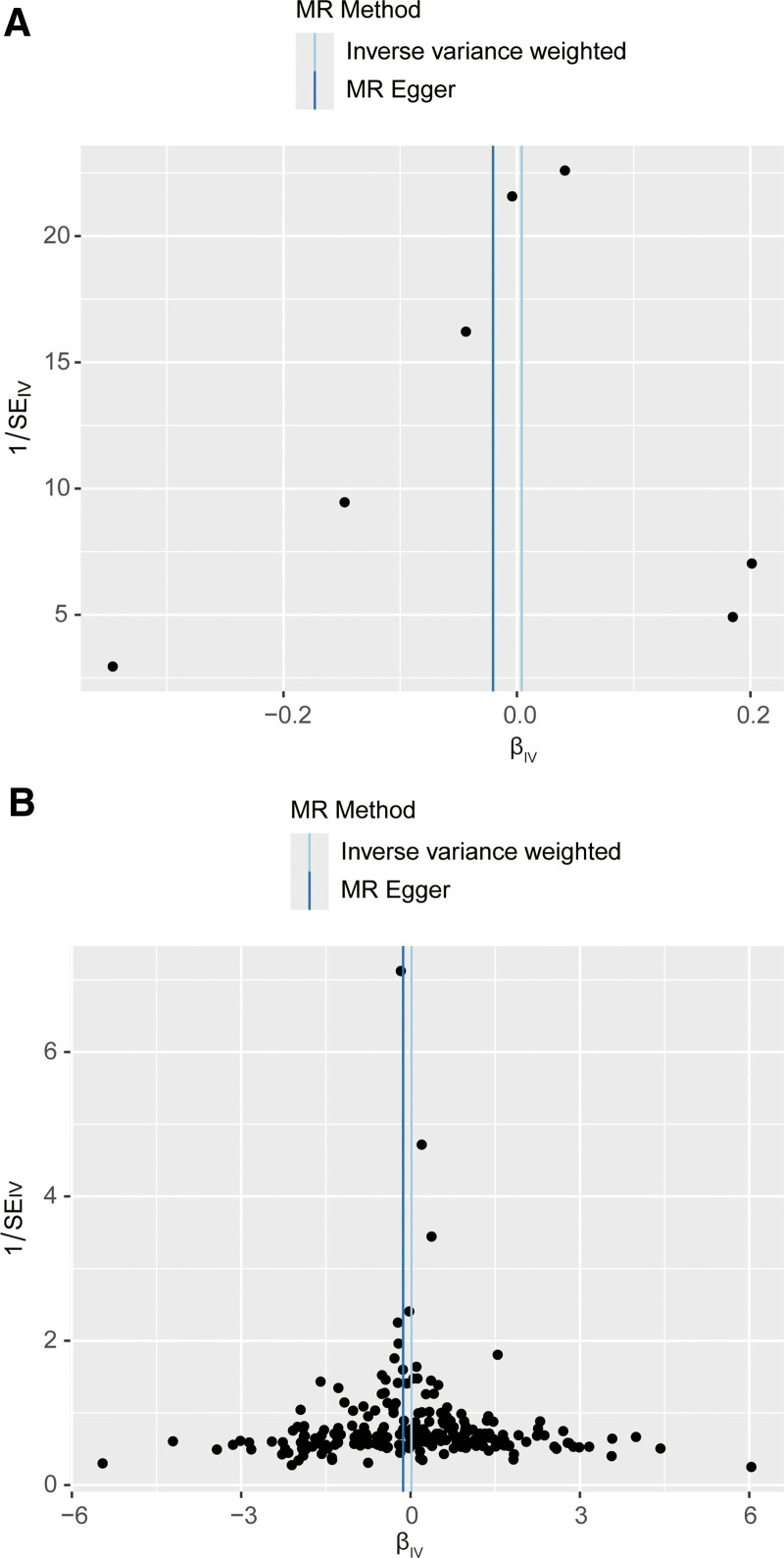
Funnel plot for IVs; each point represents 1 SNP and a uniform distribution on both sides indicates a small heterogeneity. (A) Funnel plots for MR analysis of the causal effect of gout on ED. (B) Funnel plots for MR analysis of the causal effect of serum UA levels on ED. ED = erectile dysfunction, IVs = instrumental variables, MR = Mendelian randomization, SNP = single-nucleotide polymorphism, UA = uric acid.

In this context, several MR methods were employed, and the findings indicated a lack of compelling evidence to support a causal effect of gout on ED. (IVW: OR = 1.0004, 95% confidence interval (CI) = 0.948–1.063, *P* = .888, Fig. 2A and Table 2). The MR results showed demonstrated directions of effect for across IVW, IVW, WM, WM, simple median (Fig. 2A and Table 2). There no heterogeneity no detected using *Q*-test (*P* = .232 of MR-Egger; *P* = .306 of IVW, Table 3) and funnel plot (Fig. 3A). The MR-Egger intercept test found revealed evidence of directed horizontal pleiotropy (intercept = 0.014, se = 0.029, *P* = .652, Table 3). leave-one-out plot shows illustrates the genetically predicted causal relationship between gout and ED is essentially not significantly by any single SNP (Figure S1, Supplemental Digital Content, http://links.lww.com/MD/O439). This observation underscores the robustness of our results.

**Table 2:** Mendelian randomization estimates of exposure on outcome variables.

Exposure	Outcome	Methods	Beta	OR (95% CI)	*P*-value
Gout	ED	MR-Egger	−0.020	0.980 (0.871 − 1.102)	.747
		Weighted median	0.006	1.006 (0.941 − 1.076)	.855
		Inverse variance weighted	0.004	1.004 (0.948 − 1.063)	.888
		Simple mode	0.016	1.017 (0.928 − 1.114)	.737
		Weighted mode	0.004	1.004 (0.944 − 1.069)	.898
Serum uric acid levels	ED	MR-Egger	−0.132	0.877 (0.717 − 1.073)	.203
		Weighted median	−0.143	0.867 (0.698 − 1.077)	.198
		Inverse variance weighted	0.013	1.013 (0.890 − 1.152)	.849
		Simple mode	0.250	1.284 (0.775 − 2.126)	.333
		Weighted mode	−0.049	0.952 (0.784 − 1.155)	.618

CI = confidence interval, ED = erectile dysfunction, IVW = inverse variance-weighted, MR = Mendelian randomization, OR = odds ratio, SNP = single-nucleotide polymorphism.

**Table 3:** Heterogeneity and pleiotropy tests of MR.

Exposure							Pleiotropy test		
	MR-Egger			IVW					
	*Q*	df	*Q*_*P*-value	*Q*	df	*Q*_*P*-value	Egger_intercept	SE	*P*-value
Gout	6.853	5	.232	7.168	6	.306	0.014	0.029	.652
Serum uric acid levels	192.556	222	.924	195.868	223	.905	0.005	0.003	.070

IVW = inverse variance–weighted, MR = Mendelian randomization.

### 3.3. Causal effects of serum uric acid levels on ED

The results of the MR analysis indicated that there was no increased risk of ED associated with serum UA levels. Similarly, the findings from the 5 MR analyses demonstrated that serum UA levels were not linked to an increased risk of ED (all *P* > .05, Fig. 2B and Table 2). Furthermore, Cochran *Q*-test revealed no heterogeneity (*P* = .924 of MR-Egger; *P* = .905 of IVW, Table 3), and the funnel plot did not exhibit any discernible asymmetry (Fig. 3B). MR-Egger regression was employed to assess the pleiotropy of IVs. The MR-Egger regression results indicated that the IVs exhibited no pleiotropy (intercept: 0.005, se: 0.003, *P* = .07, Table 3). The leave-one-out analysis found no influential IVs affecting the association between serum UA levels and ED (Figure S2, Supplemental Digital Content, http://links.lww.com/MD/O439).

## 4. Discussion

In this research, we performed a 2-sample MR analysis to examine the possible causal relationship between gout or serum UA concentrations and the risk of ED. Nevertheless, our results did not reveal a significant causal association between gout or serum UA levels and the incidence of ED.

The majority of current studies examining the relationship between gout and ED concentrate on the correlation between hyperuricemia and ED, as well as the association between gout and ED. Most available research indicates that both gout and elevated UA levels are significant risk factors for ED. A population-based study conducted by Schlesinger et al demonstrated that gout is associated with an increased risk of ED, thereby supporting the hypothesis that hyperuricemia may act as an independent risk factor for ED.^[19]^ A cohort study from Taiwan revealed that the hazard ratio for developing ED was 1.21 times higher (95% CI = 1.03–1.44) in individuals with gout compared to those without. The incidence of ED was found to increase with age and was notably higher in the gout group than in the non-gout group.^[20]^ Similarly, a cohort study from the United Kingdom indicated that patients with gout were 31% more likely to seek medical consultation for ED compared to those without gout, and they exhibited a significantly elevated risk of developing ED.^[21]^ Furthermore, Aribas et al identified uric acid (UA) as an independent determinant of ED.^[22]^ Concurrently, 2 recent meta-analyses demonstrated that the risk of ED in patients with hyperuricemia is 1.59 times greater than in those without hyperuricemia. Additionally, the implementation of urate-lowering therapy in patients with hyperuricemia can reduce the risk of ED by 27%. Hyperuricemia serves as a marker for systemic metabolic disorders that adversely affect erectile function.^[23,24]^

These findings from observational and meta-analysis studies conflict with those of our MR study on the association between serum gout and UA levels and ED risk. Most ED studies rely on the International Index of Erectile Function or the male sexual health questionnaire rather than penile ultrasound. Each method has limitations, resulting in inconsistent diagnostic criteria for ED.^[25]^ Additionally, all existing studies are observational, making it challenging to establish a causal relationship between gout, serum UA levels, and ED using only observational data.

The current study identifies several potential factors that may contribute to the observed association between gout and ED. Firstly, a poor psychological status may be a crucial factor in the pathogenesis of ED. Depression, as a psychogenic condition, was previously considered to be directly associated with ED.^[26]^ However, Gu et al showed that the decrease in serum UA promoted the development of depression.^[27]^ Furthermore, psychological factors are not only concomitant symptoms of gout but also risk factors for ED. Secondly, the process of penile erection is contingent upon the presence of an intact endothelium, and endothelial dysfunction represents a significant contributing factor to the development of ED.^[28]^ However, high levels of UA may contribute to endothelial recovery,^[29,30]^ which may benefit erectile function in part by protecting endothelial cells through its powerful antioxidant effect. In addition, hyperuricemia and metabolic syndrome are closely related, and metabolic syndrome itself is also associated with ED,^[31]^ so it does not prove that serum UA is an independent risk factor for ED. In a study of 3810 participants (1093 individuals with ED and 2717 individuals without ED), no significant association was observed between UA and ED (OR = 1.02, 95% CI = 0.84–1.24), and no significant differences were noted among the various UA levels (*P* = .5).^[32]^ Meanwhile, Abdul et al found that gout patients had a higher risk of developing ED in the year before diagnosis compared with non-gout patients (relative rate = 1.63, 95% CI = 1.27–2.08).^[21]^

The relationship between gout or serum UA and ED remains inconclusive. To further elucidate the direct causal relationship between gout and serum UA and ED, we employed the MR method, which revealed that gout and serum UA do not directly elevate the risk of ED in our study.

Compared to previous observational studies, MR analysis can effectively reduce potential biases including confounding factors and reverse causality, thereby strengthening causal inference. Secondly, the robustness of the MR analysis results was confirmed by sensitivity analyses and multipotency tests utilizing a variety of MR methods. It is important, however, to note the study’s limitations. Firstly, it is noteworthy that the GWAS data used in this study were derived from a European population, which may limit the generalizability of our findings to other populations. Secondly, it should be noted that this study did not distinguish between different subtypes of ED (organic or nonorganic). Future studies could focus on analyzing ED in different subgroups. Moreover, despite the application of a rigorous matching threshold to the GWAS database to eliminate potential confounding factors and associated horizontal pleiotropy, it is not possible to fully eliminate the impact of horizontal pleiotropy given many genetic variants’ unknown precise biological purposes.

## 5. Conclusion

This is the first study to explore the causal relationship between serum UA levels, gout, and ED. We did not find a causal relationship between serum UA levels or gout and ED in European populations. To a certain degree, our study ruled out the possibility that serum UA levels and gout contribute to the development of ED genetically.

## Acknowledgments

We would like to thank the participants and investigators of the database we used in this study.

## Author contributions

**Conceptualization:** Jianhua Zhang, Lei Ji.

**Data curation:** Jianhua Zhang, Lei Ji.

**Formal analysis:** Jianhua Zhang, Lei Ji.

**Funding acquisition:** Jianhua Zhang, Lei Ji.

**Investigation:** Jianhua Zhang, Lei Ji.

**Methodology:** Jianhua Zhang, Lei Ji.

**Project administration:** Jianhua Zhang, Lei Ji.

**Resources:** Jianhua Zhang, Lei Ji.

**Software:** Jianhua Zhang, Lei Ji.

**Supervision:** Jianhua Zhang, Lei Ji.

**Validation:** Jianhua Zhang, Lei Ji.

**Visualization:** Jianhua Zhang, Lei Ji.

**Writing – original draft:** Jianhua Zhang, Lei Ji.

**Writing – review & editing:** Jianhua Zhang, Lei Ji.

## Supplementary Material


