# Supplementary material for: Exploring the causal associations of gout and serum uric acid levels on erectile dysfunction: A Mendelian randomization study
Source: Medicine (Baltimore). 2025 Feb 21;104(8):e41679. doi: 10.1097/MD.0000000000041679 (PMC11856936; doi:10.1097/MD.0000000000041679)

Supplementary Table S1 Detailed information of SNPs

| Summary of genetic variants (n=7) used to estimate the effect of gout on ED in MR analysis. | | | | | | | | |
| --- | --- | --- | --- | --- | --- | --- | --- | --- |
| Chr | SNP | effect_allele | other_allele | beta.exposure | se.exposure | pval.exposure | R2 | F |
| 1 | rs1064257 | G | C | 1.0269 | 0.0913 | 2.55329E-29 | 0.00083822 | 126.505379 |
| 2 | rs112395288 | T | C | 0.7483 | 0.0981 | 2.43501E-14 | 0.0003857 | 58.1845548 |
| 3 | rs149136965 | C | G | 0.3958 | 0.0711 | 2.62102E-08 | 0.00020546 | 30.9889327 |
| 4 | rs34004016 | C | T | -0.161 | 0.0278 | 7.05602E-09 | 0.00022237 | 33.5394858 |
| 5 | rs3775948 | C | G | 0.3616 | 0.0302 | 4.8095E-33 | 0.00094981 | 143.363039 |
| 6 | rs45499402 | C | G | 0.7005 | 0.0509 | 5.03037E-43 | 0.00125442 | 189.397811 |
| 7 | rs4645896 | A | G | 0.4085 | 0.0655 | 4.47703E-10 | 0.00025787 | 38.8951779 |
| Summary of genetic variants (n=224) used to estimate the effect of Serum uric acid levels on ED in MR analysis. | | | | | | | | |
| 1 | rs10075612 | T | G | 0.0139 | 0.0023 | 1.03901E-09 | 0.00010621 | 36.523417 |
| 2 | rs10082474 | G | A | -0.0131 | 0.0018 | 1.06096E-13 | 0.00015402 | 52.9657413 |
| 3 | rs10196697 | A | G | -0.0105 | 0.0019 | 2.712E-08 | 8.8814E-05 | 30.5399886 |
| 4 | rs10211562 | T | G | -0.0134 | 0.0018 | 4.30725E-14 | 0.00016115 | 55.4194307 |
| 5 | rs1021409 | G | T | 0.0188 | 0.0024 | 4.1783E-15 | 0.00017843 | 61.3607542 |
| 6 | rs10279504 | A | C | -0.0122 | 0.002 | 9.33706E-10 | 0.00010821 | 37.2097836 |
| 7 | rs10405423 | A | C | 0.0216 | 0.002 | 2.12618E-26 | 0.00033912 | 116.639322 |
| 8 | rs10429294 | T | C | 0.0205 | 0.0018 | 4.10488E-30 | 0.00037709 | 129.706036 |
| 9 | rs10771025 | G | C | -0.0118 | 0.002 | 7.17398E-09 | 0.00010123 | 34.8097975 |
| 10 | rs10859925 | A | G | 0.0159 | 0.0023 | 1.21311E-11 | 0.00013897 | 47.7898921 |
| 11 | rs10868758 | C | T | 0.0164 | 0.0028 | 2.718E-09 | 9.9765E-05 | 34.3059229 |
| 12 | rs10886117 | A | G | 0.021 | 0.0022 | 5.01649E-22 | 0.00026493 | 91.1151725 |
| 13 | rs10899125 | G | T | 0.0226 | 0.0041 | 4.17398E-08 | 8.8361E-05 | 30.3841183 |
| 14 | rs10901057 | G | C | 0.0172 | 0.0027 | 2.36102E-10 | 0.00011801 | 40.5813826 |
| 15 | rs10922199 | A | G | 0.0106 | 0.0019 | 1.84098E-08 | 9.0514E-05 | 31.1244727 |
| 16 | rs11056306 | A | G | -0.0108 | 0.0018 | 1.17301E-09 | 0.00010469 | 35.9997906 |
| 17 | rs11056396 | C | T | -0.0192 | 0.0029 | 3.83884E-11 | 0.00012747 | 43.8332765 |
| 18 | rs11109717 | C | T | -0.0137 | 0.002 | 2.17721E-11 | 0.00013645 | 46.9222271 |
| 19 | rs11128603 | G | A | -0.0203 | 0.003 | 9.37994E-12 | 0.00013315 | 45.7875114 |
| 20 | rs11163481 | T | G | 0.0125 | 0.0018 | 1.24394E-11 | 0.00014024 | 48.2250281 |
| 21 | rs11164916 | C | G | 0.0188 | 0.0018 | 1.79184E-25 | 0.00031716 | 109.085785 |
| 22 | rs11202328 | T | C | -0.0254 | 0.0023 | 2.11495E-27 | 0.00035457 | 121.957703 |
| 23 | rs112214565 | A | G | 0.0578 | 0.0083 | 4.33711E-12 | 0.00014102 | 48.4950003 |
| 24 | rs11243143 | A | G | -0.0353 | 0.0021 | 5.12507E-63 | 0.00082111 | 282.558447 |
| 25 | rs11264341 | T | C | -0.0352 | 0.0018 | 1.56783E-82 | 0.00111098 | 382.417529 |
| 26 | rs114158982 | G | C | 0.0584 | 0.0077 | 4.94425E-14 | 0.00016727 | 57.5230252 |
| 27 | rs114165349 | C | G | 0.0805 | 0.0067 | 8.01124E-33 | 0.00041967 | 144.357592 |
| 28 | rs11564722 | T | C | -0.0204 | 0.002 | 1.45312E-24 | 0.00030249 | 104.039395 |
| 29 | rs11657044 | C | T | 0.0268 | 0.0022 | 1.18905E-34 | 0.00043141 | 148.395831 |
| 30 | rs11667586 | C | T | 0.0181 | 0.0026 | 4.917E-12 | 0.00014093 | 48.4627359 |
| 31 | rs1171614 | C | T | 0.0563 | 0.0024 | 1.7298E-120 | 0.0015979 | 550.290202 |
| 32 | rs11827106 | A | G | -0.0124 | 0.0022 | 3.32997E-08 | 9.2386E-05 | 31.7684103 |
| 33 | rs11871152 | G | A | 0.0105 | 0.0018 | 4.67197E-09 | 9.8955E-05 | 34.0275798 |
| 34 | rs11940694 | G | A | 0.0106 | 0.0018 | 3.68197E-09 | 0.00010085 | 34.6788106 |
| 35 | rs12070208 | C | T | -0.0211 | 0.0038 | 3.15697E-08 | 8.9662E-05 | 30.8315381 |
| 36 | rs12096443 | T | C | -0.0174 | 0.0019 | 1.53993E-19 | 0.00024386 | 83.8665482 |
| 37 | rs12105304 | G | A | 0.0206 | 0.0021 | 7.90679E-22 | 0.00027978 | 96.2261976 |
| 38 | rs12158162 | G | A | -0.0212 | 0.0037 | 1.196E-08 | 9.5472E-05 | 32.8296118 |
| 39 | rs12277177 | G | A | 0.0157 | 0.0027 | 4.115E-09 | 9.8328E-05 | 33.8118747 |
| 40 | rs12467636 | G | A | 0.013 | 0.0021 | 3.58699E-10 | 0.00011144 | 38.3217726 |
| 41 | rs12543287 | C | G | -0.0115 | 0.0018 | 3.84601E-10 | 0.0001187 | 40.8176638 |
| 42 | rs12575710 | A | G | 0.0264 | 0.0024 | 4.16102E-29 | 0.00035179 | 120.999296 |
| 43 | rs12666237 | T | C | 0.0202 | 0.003 | 3.03529E-11 | 0.00013184 | 45.3375141 |
| 44 | rs12708477 | C | A | -0.0124 | 0.0022 | 1.98098E-08 | 9.2386E-05 | 31.7684103 |
| 45 | rs12767261 | A | G | 0.0166 | 0.0029 | 6.67006E-09 | 9.5286E-05 | 32.7655645 |
| 46 | rs12775853 | A | T | -0.0146 | 0.0022 | 2.10717E-11 | 0.00012807 | 44.0410661 |
| 47 | rs12790943 | T | C | -0.014 | 0.0021 | 8.70563E-12 | 0.00012924 | 44.4441859 |
| 48 | rs12806743 | T | G | -0.0138 | 0.0021 | 9.56093E-11 | 0.00012558 | 43.1834223 |
| 49 | rs12887732 | A | C | 0.0114 | 0.002 | 8.44501E-09 | 9.4484E-05 | 32.489811 |
| 50 | rs12891886 | C | G | 0.013 | 0.0021 | 3.23303E-10 | 0.00011144 | 38.3217726 |
| 51 | rs12937692 | A | G | 0.0131 | 0.002 | 1.48799E-10 | 0.00012476 | 42.9022504 |
| 52 | rs12973608 | C | A | -0.014 | 0.0019 | 1.99022E-13 | 0.00015788 | 54.293313 |
| 53 | rs12979148 | C | T | 0.0149 | 0.0025 | 3.48201E-09 | 0.0001033 | 35.5213934 |
| 54 | rs12987661 | C | T | -0.0302 | 0.0029 | 4.21502E-25 | 0.0003153 | 108.446456 |
| 55 | rs12992672 | A | G | 0.0156 | 0.0024 | 2.09802E-10 | 0.00012286 | 42.2497542 |
| 56 | rs13021972 | T | A | 0.016 | 0.0018 | 4.49676E-18 | 0.00022974 | 79.0118861 |
| 57 | rs1317983 | C | T | 0.0304 | 0.002 | 2.64728E-50 | 0.0006715 | 231.038656 |

| 58 | rs13221758 | C | A | 0.0151 | 0.002 | 9.52796E-14 | 0.00016576 | 57.0021684 |
| --- | --- | --- | --- | --- | --- | --- | --- | --- |
| 59 | rs13230509 | C | G | 0.0314 | 0.0019 | 2.61216E-58 | 0.0007937 | 273.117525 |
| 60 | rs13247874 | T | C | -0.0345 | 0.0023 | 1.99022E-49 | 0.00065395 | 224.998691 |
| 61 | rs13411042 | A | C | 0.0145 | 0.0018 | 4.33012E-16 | 0.00018869 | 64.8915978 |
| 62 | rs13418518 | T | A | 0.0366 | 0.0058 | 2.22101E-10 | 0.0001158 | 39.8202202 |
| 63 | rs140753856 | G | A | -0.0147 | 0.0025 | 6.14497E-09 | 0.00010054 | 34.5741989 |
| 64 | rs140885868 | G | A | -0.0893 | 0.0097 | 2.40381E-20 | 0.00024643 | 84.7533597 |
| 65 | rs142929199 | G | A | -0.0527 | 0.0074 | 8.05935E-13 | 0.00014748 | 50.7171995 |
| 66 | rs144188351 | C | G | 0.0111 | 0.002 | 0.000000016 | 8.9577E-05 | 30.8023208 |
| 67 | rs145157727 | A | G | -0.0518 | 0.0082 | 2.97701E-10 | 0.00011605 | 39.9051813 |
| 68 | rs146787580 | C | A | 0.0264 | 0.0032 | 2.5142E-16 | 0.00019791 | 68.0621041 |
| 69 | rs148179165 | A | C | 0.0164 | 0.0019 | 3.20406E-18 | 0.00021664 | 74.5037218 |
| 70 | rs150147865 | T | A | -0.1163 | 0.0116 | 8.70162E-24 | 0.00029226 | 100.517326 |
| 71 | rs1511299 | C | T | -0.0211 | 0.002 | 4.14572E-26 | 0.0003236 | 111.301853 |
| 72 | rs152197 | T | C | -0.0126 | 0.0022 | 7.23303E-09 | 9.539E-05 | 32.8014621 |
| 73 | rs157512 | C | T | -0.0202 | 0.0025 | 7.68245E-16 | 0.00018984 | 65.2860202 |
| 74 | rs1622987 | A | G | 0.011 | 0.002 | 4.53701E-08 | 8.797E-05 | 30.249824 |
| 75 | rs16942751 | A | C | 0.0176 | 0.0027 | 3.2107E-11 | 0.00012356 | 42.4908365 |
| 76 | rs17013743 | G | A | -0.0298 | 0.0034 | 8.65964E-19 | 0.00022337 | 76.8196224 |
| 77 | rs17024258 | T | C | 0.036 | 0.0064 | 2.123E-08 | 9.2014E-05 | 31.640441 |
| 78 | rs17050272 | A | G | 0.0218 | 0.0018 | 1.58818E-34 | 0.00042641 | 146.678159 |
| 79 | rs1719985 | T | C | -0.0118 | 0.0018 | 1.59901E-10 | 0.00012497 | 42.9750587 |
| 80 | rs17473411 | C | T | 0.0174 | 0.0027 | 7.55266E-11 | 0.00012077 | 41.5306226 |
| 81 | rs17592117 | C | T | 0.0426 | 0.0031 | 1.79597E-43 | 0.00054892 | 188.839692 |
| 82 | rs17624477 | C | T | 0.0252 | 0.0038 | 5.10035E-11 | 0.00012789 | 43.9775835 |
| 83 | rs17632159 | C | G | -0.0299 | 0.0019 | 3.09671E-54 | 0.00071973 | 247.646759 |
| 84 | rs17786744 | G | A | 0.0223 | 0.0018 | 1.16091E-33 | 0.00044619 | 153.483675 |
| 85 | rs181673 | C | A | 0.0173 | 0.0018 | 1.56495E-21 | 0.00026858 | 92.3729195 |
| 86 | rs187355703 | G | C | 0.0563 | 0.0064 | 1.31795E-18 | 0.00022501 | 77.3845596 |
| 87 | rs1925258 | A | T | -0.0124 | 0.0019 | 5.4275E-11 | 0.00012386 | 42.59255 |
| 88 | rs1955949 | C | T | -0.0137 | 0.002 | 2.39497E-11 | 0.00013645 | 46.9222271 |
| 89 | rs2012385 | A | G | -0.0181 | 0.0021 | 2.71894E-17 | 0.00021601 | 74.2875497 |
| 90 | rs2039424 | A | G | 0.0111 | 0.0018 | 1.65101E-09 | 0.00011059 | 38.0275566 |
| 91 | rs2057291 | G | A | -0.0146 | 0.0019 | 5.74513E-15 | 0.0001717 | 59.046748 |
| 92 | rs2060824 | C | T | 0.0135 | 0.0018 | 3.27793E-14 | 0.00016357 | 56.2496728 |
| 93 | rs2075252 | C | T | -0.0261 | 0.002 | 1.69083E-40 | 0.00049506 | 170.301509 |
| 94 | rs2108093 | G | A | 0.0151 | 0.0023 | 1.18599E-10 | 0.00012534 | 43.1018287 |
| 95 | rs2195525 | T | C | -0.014 | 0.0019 | 6.72048E-14 | 0.00015788 | 54.293313 |
| 96 | rs219777 | A | G | -0.0227 | 0.0023 | 4.18986E-22 | 0.00028322 | 97.407751 |
| 97 | rs2210315 | T | C | 0.0181 | 0.0027 | 2.26986E-11 | 0.00013068 | 44.9393819 |
| 98 | rs2219647 | A | G | 0.0163 | 0.002 | 1.24595E-15 | 0.00019314 | 66.4221136 |
| 99 | rs2229357 | A | G | -0.0449 | 0.0022 | 1.11995E-90 | 0.00120996 | 416.528569 |
| 100 | rs2231145 | C | T | -0.0787 | 0.0046 | 3.35429E-64 | 0.00085058 | 292.705764 |
| 101 | rs2240193 | A | C | -0.0258 | 0.0037 | 2.09315E-12 | 0.00014139 | 48.6220693 |
| 102 | rs2240390 | C | T | -0.0132 | 0.0019 | 7.32487E-12 | 0.00014036 | 48.2656472 |
| 103 | rs2287084 | T | G | 0.0124 | 0.0021 | 6.35697E-09 | 0.00010139 | 34.8660103 |
| 104 | rs2362515 | A | G | -0.0121 | 0.0019 | 2.30202E-10 | 0.00011794 | 40.5565508 |
| 105 | rs2414064 | A | G | -0.0144 | 0.0025 | 7.09594E-09 | 9.6483E-05 | 33.177407 |
| 106 | rs2436958 | C | T | -0.0188 | 0.0024 | 1.44311E-15 | 0.00017843 | 61.3607542 |
| 107 | rs2437817 | A | C | -0.0146 | 0.0019 | 2.59597E-14 | 0.0001717 | 59.046748 |
| 108 | rs2480714 | T | G | 0.0126 | 0.0022 | 5.87205E-09 | 9.539E-05 | 32.8014621 |
| 109 | rs2493121 | A | T | 0.0121 | 0.002 | 1.05099E-09 | 0.00010644 | 36.6022871 |
| 110 | rs255749 | A | G | 0.0159 | 0.002 | 1.50383E-15 | 0.00018378 | 63.2021324 |
| 111 | rs2581824 | A | C | 0.03 | 0.0018 | 4.93856E-64 | 0.00080723 | 277.776162 |
| 112 | rs2636590 | A | G | 0.011 | 0.0018 | 2.28702E-09 | 0.0001086 | 37.3454618 |
| 113 | rs2668423 | G | T | -0.0115 | 0.002 | 1.085E-08 | 9.6149E-05 | 33.0623077 |
| 114 | rs2695580 | A | G | 0.0107 | 0.0019 | 1.32501E-08 | 9.2229E-05 | 31.714497 |
| 115 | rs2714345 | A | G | -0.0122 | 0.0021 | 1.388E-08 | 9.8149E-05 | 33.7503706 |
| 116 | rs272889 | G | A | 0.013 | 0.0018 | 1.85183E-12 | 0.00015168 | 52.1601904 |
| 117 | rs2749005 | G | T | 0.0116 | 0.002 | 4.66702E-09 | 9.7828E-05 | 33.6398043 |
| 118 | rs2762353 | G | A | 0.0668 | 0.0019 | 1E-200 | 0.00358209 | 1236.07037 |
| 119 | rs2788144 | G | A | 0.0395 | 0.0054 | 3.37987E-13 | 0.00015559 | 53.5062045 |
| 120 | rs2808500 | G | A | -0.0104 | 0.0018 | 5.89495E-09 | 9.708E-05 | 33.3825219 |
| 121 | rs2834317 | A | G | 0.0192 | 0.0027 | 4.9136E-13 | 0.00014705 | 50.5676071 |
| 122 | rs28419182 | A | G | 0.018 | 0.002 | 2.8721E-19 | 0.00023552 | 80.9995288 |
| 123 | rs28517717 | T | C | -0.0218 | 0.0019 | 9.77687E-30 | 0.00038273 | 131.644664 |
| 124 | rs28755272 | C | T | -0.0174 | 0.002 | 6.3387E-18 | 0.00022009 | 75.6895597 |
| 125 | rs2926590 | A | C | 0.0193 | 0.0023 | 5.1027E-17 | 0.00020475 | 70.4135791 |

| 126 | rs2941484 | T | C | 0.0318 | 0.0018 | 1.30287E-71 | 0.00090691 | 312.109296 |
| --- | --- | --- | --- | --- | --- | --- | --- | --- |
| 127 | rs2943645 | T | C | 0.0134 | 0.002 | 2.96483E-11 | 0.00013054 | 44.8897389 |
| 128 | rs2973444 | C | T | -0.0245 | 0.0036 | 1.073E-11 | 0.00013468 | 46.315317 |
| 129 | rs3116068 | T | C | 0.0184 | 0.0022 | 1.67494E-17 | 0.0002034 | 69.9500063 |
| 130 | rs3212198 | T | C | -0.0172 | 0.0018 | 4.79844E-21 | 0.00026549 | 91.3081109 |
| 131 | rs33938520 | T | C | -0.0135 | 0.0023 | 9.78701E-09 | 0.00010019 | 34.4515954 |
| 132 | rs34381009 | G | A | -0.0387 | 0.0023 | 2.09701E-61 | 0.00082273 | 283.115555 |
| 133 | rs34555420 | T | G | 0.0531 | 0.0034 | 1.40185E-55 | 0.00070888 | 243.909481 |
| 134 | rs34868798 | C | T | -0.0141 | 0.0024 | 7.8701E-09 | 0.00010037 | 34.5154242 |
| 135 | rs35523212 | G | A | -0.0448 | 0.0018 | 7.6384E-130 | 0.00179837 | 619.453187 |
| 136 | rs35994853 | T | C | 0.0151 | 0.0019 | 1.43913E-15 | 0.00018366 | 63.1602974 |
| 137 | rs3734537 | A | C | 0.0401 | 0.003 | 5.28689E-41 | 0.00051936 | 178.666739 |
| 138 | rs3824359 | C | T | 0.0142 | 0.0023 | 1.53402E-09 | 0.00011085 | 38.1169806 |
| 139 | rs3845534 | A | G | 0.0151 | 0.0018 | 3.56862E-16 | 0.00020463 | 70.3730474 |
| 140 | rs4476815 | C | G | 0.0366 | 0.0058 | 2.28502E-10 | 0.0001158 | 39.8202202 |
| 141 | rs45487598 | A | G | 0.0184 | 0.0032 | 7.703E-09 | 9.6149E-05 | 33.0623077 |
| 142 | rs45499402 | C | G | 0.1462 | 0.0024 | 1E-200 | 0.01067724 | 3710.81869 |
| 143 | rs455213 | C | T | 0.0165 | 0.0018 | 1.43714E-19 | 0.00024432 | 84.027289 |
| 144 | rs4575545 | A | G | -0.0267 | 0.0019 | 2.2269E-43 | 0.000574 | 197.475306 |
| 145 | rs4783505 | T | G | 0.0139 | 0.0019 | 6.18016E-13 | 0.00015563 | 53.5204643 |
| 146 | rs4789278 | A | G | -0.0179 | 0.0022 | 1.3471E-15 | 0.0001925 | 66.2000282 |
| 147 | rs4886755 | G | A | 0.0285 | 0.0018 | 2.83727E-57 | 0.00072858 | 250.692986 |
| 148 | rs4897160 | A | G | 0.0145 | 0.0018 | 1.7869E-16 | 0.00018869 | 64.8915978 |
| 149 | rs4966019 | T | C | -0.0323 | 0.0018 | 1.09698E-70 | 0.00093563 | 322.001213 |
| 150 | rs496708 | A | C | -0.0133 | 0.0018 | 7.26273E-13 | 0.00015876 | 54.5953614 |
| 151 | rs505870 | A | C | -0.013 | 0.002 | 1.78299E-10 | 0.00012286 | 42.2497542 |
| 152 | rs56376587 | C | A | 0.0113 | 0.0018 | 6.40605E-10 | 0.00011461 | 39.4102646 |
| 153 | rs56379622 | A | G | -0.0374 | 0.0049 | 2.89801E-14 | 0.0001694 | 58.2570539 |
| 154 | rs57577420 | G | A | 0.0187 | 0.0028 | 4.62168E-11 | 0.00012971 | 44.6030569 |
| 155 | rs589852 | T | C | -0.0288 | 0.0018 | 3.83707E-58 | 0.00074399 | 255.998511 |
| 156 | rs59864860 | A | T | -0.0169 | 0.0027 | 4.65704E-10 | 0.00011393 | 39.1780986 |
| 157 | rs6040060 | G | A | -0.0148 | 0.0023 | 1.629E-10 | 0.00012041 | 41.4061864 |
| 158 | rs60767324 | C | T | -0.0189 | 0.0033 | 0.000000012 | 9.539E-05 | 32.8014621 |
| 159 | rs6088559 | A | C | 0.0162 | 0.0021 | 1.02094E-14 | 0.00017305 | 59.5098579 |
| 160 | rs6129786 | A | C | -0.013 | 0.0021 | 3.39797E-10 | 0.00011144 | 38.3217726 |
| 161 | rs61941404 | T | C | -0.028 | 0.0037 | 2.41102E-14 | 0.00016653 | 57.2677458 |
| 162 | rs62262727 | A | G | 0.0358 | 0.004 | 3.52696E-19 | 0.00023291 | 80.1020341 |
| 163 | rs62294282 | G | A | 0.3013 | 0.0022 | 1E-200 | 0.05172899 | 18756.4384 |
| 164 | rs62294340 | A | G | -0.017 | 0.0018 | 9.99309E-21 | 0.00025935 | 89.197012 |
| 165 | rs62409885 | T | C | 0.0615 | 0.0023 | 5.3456E-164 | 0.00207511 | 714.976938 |
| 166 | rs62580766 | T | C | -0.0144 | 0.0026 | 4.74898E-08 | 8.9205E-05 | 30.6743778 |
| 167 | rs6429747 | T | C | -0.0215 | 0.0022 | 9.59622E-23 | 0.00027769 | 95.5056428 |
| 168 | rs6492910 | A | G | -0.0133 | 0.002 | 2.02209E-11 | 0.0001286 | 44.2222428 |
| 169 | rs6674490 | A | C | -0.0123 | 0.0018 | 9.67609E-12 | 0.00013579 | 46.6941728 |
| 170 | rs6715785 | A | G | -0.0116 | 0.0021 | 3.97997E-08 | 8.8733E-05 | 30.5122942 |
| 171 | rs676015 | C | T | -0.015 | 0.0019 | 1.87802E-15 | 0.00018124 | 62.3265073 |
| 172 | rs6804946 | C | T | -0.0216 | 0.0025 | 1.77296E-17 | 0.00021706 | 74.6491658 |
| 173 | rs6825697 | C | G | 0.0365 | 0.0038 | 1.11712E-21 | 0.00026826 | 92.2605437 |
| 174 | rs6860575 | T | C | -0.0114 | 0.0018 | 1.88499E-10 | 0.00011664 | 40.1108778 |
| 175 | rs686364 | G | A | 0.0167 | 0.002 | 3.09671E-17 | 0.00020274 | 69.7220944 |
| 176 | rs6939175 | A | G | -0.012 | 0.002 | 3.596E-09 | 0.00010469 | 35.9997906 |
| 177 | rs7093087 | A | G | 0.0172 | 0.0024 | 4.3481E-13 | 0.00014935 | 51.3608124 |
| 178 | rs7122026 | A | C | 0.0121 | 0.002 | 1.39499E-09 | 0.00010644 | 36.6022871 |
| 179 | rs7188156 | G | T | 0.0276 | 0.0024 | 2.51884E-31 | 0.00038448 | 132.249231 |
| 180 | rs7200986 | A | G | -0.0135 | 0.0018 | 3.00193E-13 | 0.00016357 | 56.2496728 |
| 181 | rs7224610 | A | C | -0.0265 | 0.0019 | 6.51028E-44 | 0.00056544 | 194.527954 |
| 182 | rs7230932 | A | G | -0.0101 | 0.0018 | 4.09402E-08 | 9.156E-05 | 31.4843848 |
| 183 | rs7247977 | C | T | 0.0176 | 0.0018 | 2.46604E-22 | 0.00027798 | 95.6043822 |
| 184 | rs72818964 | A | G | 0.0155 | 0.0027 | 5.58895E-09 | 9.5839E-05 | 32.9559126 |
| 185 | rs72951456 | T | C | 0.0264 | 0.0048 | 3.16701E-08 | 8.797E-05 | 30.249824 |
| 186 | rs7302925 | G | A | -0.0184 | 0.0023 | 2.30409E-15 | 0.0001861 | 63.9996277 |
| 187 | rs73169739 | C | T | 0.0242 | 0.0036 | 2.90202E-11 | 0.00013141 | 45.1880088 |
| 188 | rs7331398 | C | T | -0.0148 | 0.0019 | 1.36994E-14 | 0.00017644 | 60.6755473 |
| 189 | rs73349296 | T | G | -0.0146 | 0.0022 | 4.48642E-11 | 0.00012807 | 44.0410661 |
| 190 | rs7341879 | G | A | 0.0121 | 0.0022 | 3.96004E-08 | 8.797E-05 | 30.249824 |
| 191 | rs7402939 | C | T | 0.012 | 0.0018 | 3.5229E-11 | 0.00012924 | 44.4441859 |
| 192 | rs74397112 | T | C | 0.0227 | 0.0029 | 5.19159E-15 | 0.00017817 | 61.2707494 |
| 193 | rs74606487 | G | A | -0.0148 | 0.0027 | 3.94903E-08 | 8.7379E-05 | 30.0464645 |

| 194 | rs753009 | T | C | 0.0142 | 0.0021 | 3.63162E-11 | 0.00013296 | 45.72309 |
| --- | --- | --- | --- | --- | --- | --- | --- | --- |
| 195 | rs754600 | A | G | 0.0152 | 0.0021 | 1.31009E-12 | 0.00015235 | 52.3897179 |
| 196 | rs75523587 | A | T | 0.0174 | 0.0022 | 1.94402E-15 | 0.0001819 | 62.5533552 |
| 197 | rs7642977 | T | C | 0.0106 | 0.0019 | 3.94003E-08 | 9.0514E-05 | 31.1244727 |
| 198 | rs7651369 | G | A | -0.011 | 0.0019 | 1.31E-08 | 9.7473E-05 | 33.5178106 |
| 199 | rs7677783 | T | C | -0.0114 | 0.0018 | 3.34103E-10 | 0.00011664 | 40.1108778 |
| 200 | rs7696556 | C | A | -0.0168 | 0.0023 | 2.47913E-13 | 0.00015515 | 53.3531868 |
| 201 | rs77008184 | G | A | 0.0188 | 0.0029 | 1.695E-10 | 0.00012221 | 42.0259149 |
| 202 | rs7766720 | C | T | 0.021 | 0.0029 | 6.35185E-13 | 0.00015248 | 52.4372693 |
| 203 | rs7773175 | G | C | -0.0216 | 0.002 | 6.51628E-28 | 0.00033912 | 116.639322 |
| 204 | rs784257 | C | T | 0.0149 | 0.0026 | 1.34499E-08 | 9.5507E-05 | 32.8415249 |
| 205 | rs78671965 | T | A | 0.0271 | 0.0045 | 1.73002E-09 | 0.00010547 | 36.2669495 |
| 206 | rs79141906 | C | A | -0.0123 | 0.0022 | 1.58001E-08 | 9.0902E-05 | 31.2580826 |
| 207 | rs7952306 | T | G | 0.025 | 0.0024 | 6.13903E-26 | 0.00031548 | 108.506313 |
| 208 | rs8039645 | A | C | 0.0127 | 0.0022 | 1.011E-08 | 9.691E-05 | 33.3241863 |
| 209 | rs807624 | T | G | -0.0168 | 0.0019 | 8.23E-19 | 0.00022733 | 78.1823707 |
| 210 | rs831036 | G | C | 0.0146 | 0.0018 | 7.4817E-16 | 0.0001913 | 65.7897408 |
| 211 | rs833805 | G | A | 0.0226 | 0.0028 | 1.47096E-15 | 0.00018944 | 65.1475802 |
| 212 | rs836968 | T | C | -0.014 | 0.0019 | 3.59335E-13 | 0.00015788 | 54.293313 |
| 213 | rs856560 | C | T | 0.0133 | 0.002 | 2.73527E-11 | 0.0001286 | 44.2222428 |
| 214 | rs926979 | C | T | 0.0137 | 0.002 | 3.3512E-12 | 0.00013645 | 46.9222271 |
| 215 | rs928122 | T | C | -0.0146 | 0.0023 | 2.84898E-10 | 0.00011718 | 40.2946616 |
| 216 | rs9307594 | G | A | 0.0105 | 0.0019 | 2.91803E-08 | 8.8814E-05 | 30.5399886 |
| 217 | rs9333592 | T | C | -0.0239 | 0.0039 | 5.83499E-10 | 0.00010921 | 37.5546796 |
| 218 | rs9534949 | G | C | -0.0133 | 0.0019 | 6.51178E-12 | 0.00014249 | 48.999715 |
| 219 | rs963837 | C | T | -0.0245 | 0.0018 | 4.72063E-43 | 0.00053852 | 185.261268 |
| 220 | rs9807214 | A | G | 0.0128 | 0.0021 | 1.31601E-09 | 0.00010804 | 37.1517113 |
| 221 | rs9843304 | T | C | 0.0103 | 0.0018 | 5.07201E-09 | 9.5222E-05 | 32.7436367 |
| 222 | rs9872445 | T | C | -0.0126 | 0.002 | 8.30501E-10 | 0.00011542 | 39.6897691 |
| 223 | rs9880232 | A | C | -0.0144 | 0.0022 | 3.92645E-11 | 0.00012459 | 42.842726 |
| 224 | rs9927317 | G | C | 0.0177 | 0.0021 | 1.92398E-17 | 0.00020657 | 71.0404031 |
| Chr, chromosome; SNP, Single nucleotide polymorphism; Se, standard error; ED, Erectile dysfunction. | | | | | | | | |

Supplementary figure S1. Leave-one-out analysis of the effect of gout on Erectile Dysfunction.


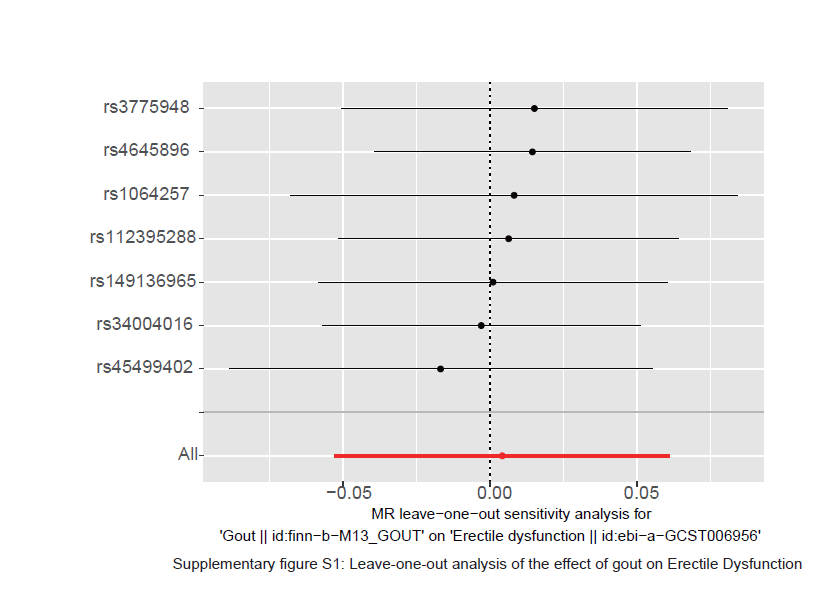


Supplementary figure S2. Leave-one-out analysis of the effect of serum uric acid levels on Erectile Dysfunction


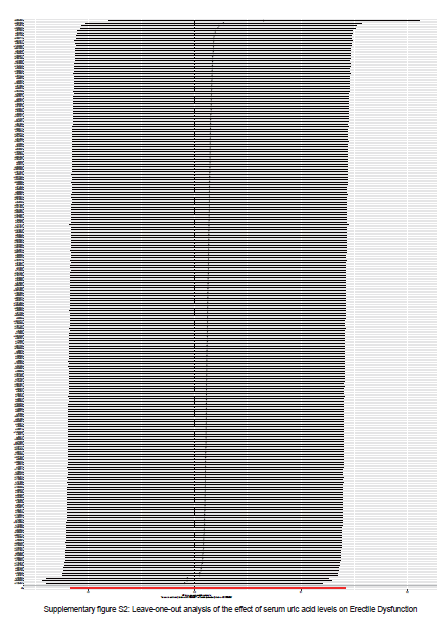

Supplement: Supplementary file 1 [file medi-104-e41679-s001.docx]
